# Evaluation of Factors That Contribute to Intraoperative and Postoperative Complications Following Colorectal Cancer Surgeries at King Abdulaziz University Hospital, Jeddah, Saudi Arabia

**DOI:** 10.7759/cureus.52339

**Published:** 2024-01-15

**Authors:** Amal A Alzahrani, Suhail A Alturkistani, Hassan Alturki, Razana S Baeisa, Jamila A Banoun, Reem A Alghamdi, Jazmin A Alghamdi

**Affiliations:** 1 Medicine, King Abdulaziz University Hospital, Jeddah, SAU; 2 Surgery, Gastrointestinal Oncology Unit, King Abdulaziz University Hospital, Jeddah, SAU

**Keywords:** general surgery and colorectal surgery, colorectal cancer surgery, intraoperative complications, post-operative complication, colorectal cancer

## Abstract

Background: Colorectal cancer (CRC) is a major contributor to cancer-related mortality and morbidity due to its high prevalence. Surgery remains the curative option. Colorectal cancer patients come to our institute at an advanced stage due to the lack of adequate national screening programs in developing countries. This carries a particularly high risk of morbidity and mortality. In this study, we aim to provide an overview of the complications of colorectal cancer surgery and to describe the preoperative and intraoperative factors associated with it.

Methods: This retrospective record review was done at King Abdulaziz University Hospital (KAUH), a tertiary center in Jeddah, Saudi Arabia. It included all patients aged 18 and older who have undergone colorectal cancer surgeries from January 2017 until August 2022.

Results: In our sample of 195 patients, 52.3% of the patients were males. The mean age of our sample was 59.32 ± 13.21. We found that 19 (9.7%) patients had an intraoperative complication (IOC). The most frequent IOC was bleeding reported in seven patients (3.6%), followed by intestinal injury in three (1.2%), bladder injury in three (1.2%), and ureter injury in three (1.2%). Regarding preoperative lab tests, patients who had low blood albumin levels (P = 0.004) and high preoperative white blood cell count (WBC; P = 0.015) were more likely to experience IOC. There was a statistically significant relationship between the patient's ASA score and IOC (P = 0.011). Postoperative complications (POC) occurred in 58 patients (29.7%). The most frequent POC was surgical site infection (SSI; 16.4%), followed by urinary tract infections (UTI) (6.7%) and prolonged postoperative ileus (5.6%). Patients who initially presented with vomiting (P = 0.015), had free air on a preoperative abdominal computed tomography (CT) scan (P = 0.028), required intraoperative blood transfusions (P = 0.033), were diagnosed with transverse colon tumors (P = 0.045), and required longer hospital stays (P = 0.011) were found to have a higher rate of POC.

Conclusion: The incidence of colorectal cancer is increasing, and surgery is a successful treatment option. However, complications from surgery may result in morbidities and prolonged hospital stays. The risk of IOC is increased by preoperative variables such as high WBC levels, low albumin, and ASA scores. Patients with initial obstruction signs, free air on CT scans, intraoperative blood transfusions, transverse colon tumors, and longer hospital stays have a higher rate of POC. Patient monitoring and the provision of standardized clinical tools enhance general survival and quality of life.

## Introduction

Colorectal cancer (CRC) is the second leading cause of cancer-related mortality and the third most frequent cancer globally, according to the International Agency for Research on Cancer (IARC) [1]. Compared to other general surgery subspecialties, colorectal surgery has a higher risk of morbidity and mortality [2]. With morbidity rates reaching 35%, the overall mortality rates after colorectal surgery range from 1 to 16.4% [3,4]. Despite the rapid advancement of immunotherapy, radiation, and chemotherapy, surgery remains the only curative treatment for advanced-stage colorectal cancer [5].

Surgical complications continue to pose a substantial concern to every surgeon because of the detrimental outcomes they can have [6]. Complications are frequently split into intraoperative complications (IOCs) and postoperative complications (POCs) [7]. Fundamental IOCs such as bleeding, intestinal injury, ureteral lesions, and bladder injuries are attributable to intra-abdominal adhesiveness, anatomic abnormality, the surgeon's experience, and many other factors [8].

POCs are known as those that occur before hospital discharge or within 30 days of the index surgery [9]. POCs of colorectal surgeries include surgical site infection (SSI), anastomotic leakage, ileus, and bowel obstruction [2].

On a global scale, a prior pooled analysis revealed that IOC incidence was 7.4%, with intestinal damage being the most common IOC at 32.1%, while POC accounted for 22%, with anastomotic leakage (27.7%) being the most frequent POC [10]. Additionally, a recent study found that up to 13% of patients who had colorectal surgery experienced wound complications like infection, hematoma, and dehiscence [3,11].

Surgical outcomes and complications are determined by complex interactions between a variety of factors, including patient characteristics, diagnosis, and type of procedure [9]. Previous research has shown that the patient's age, body mass index (BMI), nutritional state, length of operation, and surgeon expertise are all risk factors for developing POC [8,12]. Moreover, cardiovascular disease and diabetes have already been associated with an increase in postoperative morbidity; therefore, it is more crucial to check patients to enhance surgical outcomes since the colorectal cancer population is aging and has numerous concurrent conditions [13-15].

Only a few population-based studies have been done to study the associated factors of IOC and POC following surgeries of colorectal cancer patients, especially in Saudi Arabia and the Western region. In fact, little is known about the relative contribution of various factors to different outcomes in colorectal procedures. Therefore, in this study, we aim to provide an overview of the complications of colorectal cancer surgery and to describe the preoperative and intraoperative factors associated with it.

## Materials and methods

This retrospective record review was done at King Abdulaziz University Hospital (KAUH), a tertiary center in Jeddah, Saudi Arabia. All patients from the age of 18 years and older who have undergone colorectal cancer surgeries from January 2017 until August 2022 were included in this study.

All medical records of the Surgical Department of patients who have undergone colorectal cancer surgeries from January 2017 until August 2022 were reviewed, with a total of 195 patients as the sample size required for this study. The patient's data at KAUH were collected through the Phoenix database system. A predesigned checklist using Google Forms was prepared previously to collect the data. The first section included preoperative variables such as demographic characteristics like age, gender, and BMI.

The second section included data about previous medical history, including comorbidities and whether they had had any previous abdominal surgery. The third section was about the presentation of colorectal disease, including the symptoms and admitting the diagnosis.

The fourth section included questions about blood tests such as red blood cell count (RBC), white blood cell count (WBC), liver function tests (LFT), postoperative cultures, erythrocyte sedimentation rate (ESR), and C-reactive protein (CRP). The fifth section was about radiological findings such as visualization of the colon and rectum, whether they are distended, their wall thickness, any strictures, adhesions, fistulas, or signs of perforation like free intra-abdominal air.

The sixth section contained details regarding the intraoperative variables that were taken from the operation record, such as the date of the procedure, its length, and the anticipated blood loss or other IOC. As well as whether conversion to open colorectal surgery was carried out and its justification. The seventh section reviewed the histopathology findings to confirm the diagnosis and the accurate thickness of the colon and rectum. The last thing to be reviewed was the POC from the patient's postoperative notes, follow-up visits, length of hospital stay, and readmission notes.

The data were entered through Microsoft Excel 2019 (Microsoft® Corp., Redmond, WA), and IBM Statistical Package for the Social Sciences (SPSS) Statistics Version 21 (IBM Corp., Armonk, NY) was used for statistical analysis. The descriptive statistics, such as categorical variables, were described using frequencies. Other continuous variables for normally distributed data were described using means and standard deviations. As for inferential statistics, the analysis was conducted for categorical variables using the chi-square test to check for all possible risk factors. An independent T-test was used to assess relationships for normally distributed variables. Lastly, tests with a p-value < 0.05 were considered significant.

## Results

Our study aims to investigate colorectal cancer surgery complications and associated factors in KAUH, Jeddah, Saudi Arabia. In our sample of 195 patients, 52.3% were male, 47.7% were female, and 43.1% were Saudis. The mean age of our sample was 59.32±13.21. BMI data revealed that most patients are within the normal range (34.4%). Comorbidities were present in 117 (60%) of the patients, with hypertension being the most common comorbidity in 37.9% of patients, followed by diabetes mellitus in 32.8%. Of the total number of patients, 154 (79%) presented with symptoms, 63 (35.4%) had stage 3 colorectal cancer, and 15.9% received neoadjuvant chemotherapy. Further details are displayed in Table 1.

**Table 1:** Patient characteristics and demographic data. BMI: body mass index, HTN: hypertension, DM: diabetes mellitus, CKD: chronic kidney disease, IHD: ischemic heart disease, COPD: chronic obstructive pulmonary disease, IBD: irritable bowel disease, UC: ulcerative colitis.

Variables	n	%
Gender		
Female	93	47.7
Male	102	52.3
Nationality		
Saudi	84	43.1
Non-Saudi	111	56.9
BMI		
Underweight	8	4.1
Normal	67	34.4
Overweight	63	32.3
Obesity	57	29.2
Comorbidities		
	117	60
HTN	74	37.9
DM	64	32.8
Dyslipidemia	23	11.8
Anemia	15	7.7
IHD	14	7.2
Asthma	7	3.6
Anticoagulant	6	3.1
Hypothyroidism	5	2.6
CKD	2	1
Stroke	2	1
Liver diseases	2	1
COPD	1	0.5
Pre-existing IBD		
UC	3	1.5
Crohn’s disease	3	1.5
Presenting symptoms		
	154	79
Abdominal pain	82	42.1
Constipation	62	31.8
Weight loss	44	22.6
Vomiting	30	15.4
Blood in stool	27	13.8
Stage of colorectal cancer		
Stage 1	38	21.3
Stage 2	59	33.1
Stage 3	63	35.4
Stage 4	18	10.1
Neoadjuvant chemoradiation		
	31	15.9

Operative information

According to our findings, 165 (84.6%) of colorectal cancer procedures were elective, while 30 (15.4%) of the patients needed emergency surgery. The mean operation duration was 389.41±163.65 minutes. Most of the surgeries were low anterior resections (LAR), which were performed on a total of 84 patients. Laparoscopic was the most frequently used surgical approach. Furthermore, 21 patients (10.8%) required conversion from laparoscopic to open surgery, with difficult dissection being the main factor in 17 cases. Moreover, the mean of estimated intraoperative blood loss was 331.03±334.27 ml and 52 patients required blood transfusions during surgery. More details of the operative information are shown in Table 2.

**Table 2:** Operation information details. LAR: low anterior resection, APR: abdominoperineal resection.

		n (%) (total = 195)
Operation urgency	Elective	165 (84.6)
	Emergency	30 (15.4)
Operation type	LAR	84 (43.1)
	Right hemicolectomy	45 (23.1)
	APR	17 (8.7)
	Left hemicolectomy	15 (7.7)
	Subtotal colectomy	14 (7.2)
	Total colectomy	11 (5.6)
	Hartmann's procedure	9 (4.6)
ASA score	I	5 (2.6)
	II	104 (53.3)
	III	85 (43.6)
	IV	1 (0.5)
Surgical approach	Laparoscopic	133(68.2)
	Laparotomy	62 (31.8)
Conversion to open surgery	Yes	21 (10.8)
	Due to bleeding	2 (1)
	Due to difficult dissection	17 (8.8)
	Due to injury to adjacent organs	2 (1)
Need for perioperative blood transfusion	Yes	52 (26.7)
	1 packed RBC bags	37 (19)
	2 packed RBC bags	10 (5.1)
	3 packed RBC bags	4 (2.1)
	4 packed RBC bags	2 (1)

Intraoperative complications

Upon analysis of the intraoperative details, we found that 19 patients experienced IOC, resulting in an overall rate of 9.7%. The most frequent IOC was bleeding reported in seven patients (3.6%), bladder injury in three (1.5%), and ureter injury in three (1.5%). Injury to the small bowel was reported in three patients (1.5%). The least frequent IOC was an injury to the vagina reported by one person (0.5%). A demonstration of IOC is shown in Table 3.

**Table 3:** Intraoperative complications of colorectal cancer surgeries.

Intraoperative complications	n (%) 19 (9.7)
Bleeding	7 (3.6)
Bladder injury	3 (1.5)
Ureter injury	3 (1.5)
Injury to small bowel	3 (1.5)
Injury to vagina	1 (0.5)

Regarding preoperative lab tests, patients who had low blood albumin levels were more likely to experience IOC (P = 0.004). Furthermore, a high preoperative WBC count was strongly associated with an increased risk for IOC (P = 0.015). There was a statistically significant relationship between the patient's American Society of Anesthesiologists (ASA) score and IOC (P = 0.011). More details on IOC-associated variables are listed in Tables 4-5.

**Table 4:** Intraoperative complications associated variables. *Significant P < 0.05.

Variables		No IOC n (%) 176 (90.3)	IOC n (%) 19 (9.7)	P-value
Age (mean±SD)		58.824±13.111	64±13.581	0.092
Hypertension		63 (85.1)	11 (14.9)	0.102
Diabetes mellitus		57 (89.1)	7 (10.9)	0.892
Dyslipidemia		19 (82.6)	4 (17.4)	0.250
Length of hospital stay (days)		13.57±15.09	20.42±17.793	0.653
Operation duration (minutes)		387.676±163.086	405.526±172.515	0.066
Preoperative albumin (mean ± SD)		35.767±8.501	29.293±8.311	0.004*
Preoperative WBCs count (mean ± SD)		6.961±3.15	8.9±4.261	0.015*

**Table 5:** Intraoperative complications associated variables. *Significant P < 0.05.

Variables		No IOC n (%) 176 (90.3)	IOC n (%) 19 (9.7)	P-value
Sex	Male	94 (92.2)	8 (7.8)	0.487
	Female	82 (88.2)	11 (11.8)	
BMI	Underweight	8 (100)	0	0.450
	Normal	59 (88.1)	8 (11.9)	
	Overweight	55 (87.3)	8 (12.7)	
	Obese	54 (94.7)	3 (5.3)	
ASA score	I	5 (100)	0	0.011*
	II	100 (96.2)	4 (3.8)	
	III	70 (82.4)	15 (17.6)	
	IV	1 (100)	0	

Postoperative complications

In contrast to IOC, POC occurred in 58 patients (29.7%). The most frequent POC was SSI, which made up 16.4% of all POC, followed by urinary tract infection (UTI) and prolonged postoperative ileus. Table 6 displays information about POC. Only one incident of postoperative mortality has been documented, and in that case, the patient had a ruptured cecal tumor and entered septic shock immediately after the surgery. The majority of patients with POC were managed conservatively. 35 patients received conservative care. While 22 patients required surgery, the complications that required reoperations the most frequently were fascial closures, anastomotic leaks, and deep SSI.

**Table 6:** Postoperative complications of colorectal cancer surgeries. SSI: surgical site infections, UTI: urinary tract infections, DVT: deep vein thrombosis.

Postoperative complications	n (%) 58 (29.7)
SSI	32 (16.4)
UTI	13 (6.7)
Prolonged postoperative ileus	11 (5.6)
Hernia	7 (3.6)
Anastomotic leakage	5 (2.6)
Anastomotic stricture	4 (2.1)
Wound dehiscence	6 (3.1)
Sepsis	5 (2.6)
Stoma complications	4 (2.1)
Pneumonia	4 (2.1)
Pulmonary embolism	3 (1.5)
Hematoma	3 (1.5)
DVT	2 (1)

Our study found that a higher rate of POC was associated with patients who initially reported symptoms of bowel obstruction, such as vomiting (P=0.015), had free-air findings on pre-operative CT scans (P=0.028), required an intraoperative blood transfusion (P=0.033), or had been diagnosed with a transverse colon tumor (P=0.045). Patients who also required a longer hospital stay (P=0.011) were also associated with a higher rate of POC. More details regarding variables associated with POC are displayed in Tables 7-8.

**Table 7:** Postoperative complications associated variables. *Significant P < 0.05.

Variables		No POC n (%) 58 (29.7)	POC n (%) 137 (70.3)	P-value
Age (mean ± SD)		58.613±13.172	61.017±13.265	0.246
Hypertension		47 (34.3)	27 (46.6)	0.147
Diabetes mellitus		41 (29.9)	23 (39.7)	0.248
Dyslipidemia		11 (47.8)	12 (52.2)	0.024*
Length of hospital stay (days)		12.41±16.31	18.57±12.302	0.011*
Operation duration (minutes)		396.861±162	371.828±167.59	0.330
Presentation vomiting		15 (50)	15 (50)	0.015*
Free air on CT		1 (20)	4 (80)	0.028*
Tumor site in transverse colon		5 (41.7)	7 (58.3)	0.045*
Intraoperative blood transfusion		30 (57.7)	22 (42.3)	0.033*

**Table 8:** Postoperative complications-associated variables. *Significant P < 0.05.

Variables		No POC n (%) 58 (29.7)	POC n (%) 37 (70.3)	P-value
Sex	Male	77 (75.5)	25 (24.5)	0.129
	Female	60 (64.5)	33 (35.5)	
BMI	Underweight	6 (4.4)	2 (3.4)	0.807
	Normal	44 (32.1)	23 (39.7)	
	Overweight	45 (32.8)	18 (31)	
	Obese	42 (30.7)	15 (25.9)	
ASA score	I	4 (80)	1 (20)	0.032*
	II	81 (77.9)	23 (22.1)	
	III	51 (60)	34 (40)	
	IV	1 (100)	0	

Conversion

Conversion to open surgery occurred in 21 patients (10.8%). Patients who had longer operations (P=0.00), initially presented with hematochezia (P=0.01), and had radiation therapy prior to surgery (P=0.048) were more likely to convert to an open procedure. Additionally, postoperative UTI rates were higher in patients who underwent open surgery (P=0.007). Also, patients who underwent conversion had higher readmission rates (P=0.03) and higher rates of getting additional surgery due to intra-abdominal bleeding (P=0.031). Table 9 contains more information.

**Table 9:** Variables associated with conversion. *Significant P < 0.05.

Variables		No conversion n (%) 174 (89)	Conversion n (%) 21 (10.8)	P-value
Age (mean±SD)		58.891±13.195	62.952±13.101	0.184
Sex	Male	92 (52.9)	10 (47.6)	0.823
	Female	82 (47.1)	11 (52.4)	
BMI	Underweight	7 (4)	1 (4.8)	0.966
	Normal	59 (33.9)	8 (38.1)	
	Overweight	57 (32.8)	6 (28.6)	
	Obese	51 (29.3)	6 (28.6)	
Length of hospital stay (days)		14.39±16.133	13±8.019	0.698
Operation duration (minutes)		369.989±152.294	550.381±169.322	0.00*
Presentation Hematochezia		47 (27)	12 (57.1)	0.01*
UTI (POC)		8 (4.6)	5 (23.8)	0.007*
Readmission		13 (7.5)	5 (23.8)	0.03*
Reoperation due to intra-abdominal bleeding		1 (0.6)	2 (9.5)	0.031*
Radiotherapy before operation		27 (15.5)	8 (38.1)	0.048*

## Discussion

Intraoperative complications

According to our findings, 9.7% of patients had IOC. The most frequent complication was bleeding (3.6%), followed by bladder injury, ureter injury, and perforation, with a percentage of 1.2% for each. A previous study reported a 4.1% IOC consisting of bleeding (1.6%) and solid organ lesions (1.6%) [16]. Furthermore, a previous study done in Switzerland reported closer rates of 7.4% IOC, with bowel injury being the most common IOC with a percentage of 32.1% [8]. The difference in incidence rate could be related to the surgical team's experience with the operative technique, equipment, different admitting diagnoses, and if the patient has had previous abdominal surgery, which may lead to multiple intra-abdominal adhesions resulting in massive dissection intraoperatively. Moreover, the type of IOC may vary between our study and those conducted abroad and may be equivalent to those conducted locally since these variables differ from one nation to another.

The overall conversion rate in our findings was 10.8%. A study conducted in 2015 reported a conversion rate of 16.6% [17]. Furthermore, another study reported a closer conversion rate of 14.3% [18]. This range suggests that surgeons have different opinions and levels of expertise when deciding whether to convert to an open procedure, depending on the reason - difficult dissection, intraoperative bleeding, or damage to nearby organs.

Our data showed that patients with low blood albumin levels had a higher incidence of IOC, which likely will affect the length of hospital stay and the course of their recovery after surgery. As previously demonstrated in the literature, having low albumin levels before colon cancer surgery increases morbidity [3] and reduces overall survival [19]. Additionally, a high WBC count prior to surgery was associated with a higher IOC. Similarly, previous studies have shown significant links between patient morbidity and preoperative leukocytosis [20]. Quantitative laboratory tests can be used as objective measurements to help direct the management of individuals who are at a greater risk for morbidity and mortality.

Postoperative complications

Our study found a rate of POC of 29.7%, which is considerably in line with previous worldwide studies that found POC following colorectal cancer procedures occurred at a rate between 28% and 35% [3,4].

Moreover, results revealed that 16.4% of all POCs were SSI, making it the most prevalent POC. The following most frequent POCs were, respectively, UTI and prolonged postoperative ileus. According to previous studies, the most frequent complications after colorectal surgery are of an infectious nature, such as wound infections or organ space infections, as well as GI motility issues, such as ileus and bowel obstruction [2]. Furthermore, in a study done in 2017, the POC rate was found to be 17.9%. The most common surgical POC were surgical site infections, occurring in 12.1%, followed by anastomotic leaks that occurred in 3.7% of patients [21]. Following colon cancer surgery, an infection at the surgical site may increase hospital expenses and length of stay while decreasing patient quality of life. We suggest using standardized clinical evaluation procedures to help identify and monitor individuals at risk.

Our findings show an association between a higher ASA score and a higher risk for complications during and after surgery. Higher ASA scores have been shown to significantly increase the risk of IOC and POC [8]. Moreover, other studies have shown that an ASA score greater than 2 is an independent risk factor for mortality [4,22]. The ASA score identifies high-risk patients with various comorbidities, assesses their relative severity, and recommends a level of optimization. The development of consistent instruments to evaluate and categorize patients' physical status lays the foundation for improved healthcare delivery.

Intraoperative blood transfusion recipients were found to have an increased risk of developing POC. A prior study showed that blood transfusion recipients experienced a higher incidence of infectious post-operative complications [23]. Other studies showed that patients undergoing surgery for colon cancer had lower survival rates, postoperative complications, and inflammation following the procedure when a perioperative blood transfusion was performed [24]. This association could be explained by the hypothesis that allogeneic blood transfusion may reduce the host's ability to respond adaptively to infections and circulating or micrometastatic tumor cells [25].

Our data shows that POC was more common in individuals who had an abdominal CT scan with a perforation detectable by free air. A previous systematic review concluded that individuals with colon cancer who first experienced a perforation had lower overall survival rates and a greater incidence of POC [26]. This could explain why emergent colorectal cancer due to perforation is a reflection of an advanced disease that has not been identified and treated at an earlier stage; consequently, this highlights the importance of increasing public awareness regarding early colorectal cancer screening.

Furthermore, POC was linked to longer hospital stays, which was consistent with findings from another study that indicated both minor and severe complications from colorectal cancer procedures were predictive of longer hospital stays [27]. Prolonged hospital stays can have detrimental effects on patients, leading to mental and physical deconditioning and negatively influencing the healthcare system's economics. However, these effects can be mitigated by preventing complications and identifying them early.

Limitations

One of the primary limitations of this study is that it was conducted in a single medical facility, a tertiary medical center, which may not be a representative sample of the broader population. Additionally, the lack of an objective method of assessment made it impossible to evaluate the skills of the surgeons, and the experience of the surgeons greatly influences the complications that arise during and after surgery. Complications unrelated to surgery, like cardiac or respiratory issues, were not identified in enough detail because of the limited amount of information collected.

## Conclusions

The incidence of colorectal cancer has increased during the last few years. Surgery has been used successfully to remove and treat cancer; however, it has been noted that there are several intraoperative and postoperative complications that might seriously result in morbidities and extended hospital stays. Associated variables before and during surgery can predict the likelihood of these complications. Pre-operatively, low albumin, high WBC levels, and ASA scores can predict intraoperative complications. Additionally, postoperative complications can be predicted by initial symptoms of vomiting, free air on a CT scan, the requirement for intraoperative blood transfusions, and histopathological tumor length. For a higher overall survival rate and a better quality of life, it may be helpful to monitor patients and provide a standardized clinical tool to identify those who are at risk of experiencing these adverse effects.
